# Iowa State Veterinary Medical Association

**Published:** 1892-12

**Authors:** S. Stewart

**Affiliations:** Secretary


*Iowa State Veterinary Medical Association.* The fifth annual meeting of the Iowa State Veterinary Medical Association was held at Marshalltown, Iowa, November 10 and 11, 1892. The meeting was called to order by Vice-President Dr. F.H.P. Edwards. Members present: Drs. Edwards, Chatterton, Starkey, H. Shipley, L. U. Shipley, Derwent, Brown, Stalker, Reynolds, Niles, E. E. Sayers, Stewart and Green. Visitors: Drs. H. M. Replogle, J. A. Replogle, E. K. Payne, W. R. Cooper and G. A. Shipley.

The minutes of last meeting were read and approved.

Drs. Derwent and H. Shipley were appointed a committee to examine the Treasurer's accounts.

Drs. Brown, Starkey and Buffington were appointed to fill vacancies in the Board of Censors.

Verbal reports of interesting cases occupied the remaining time till noon of the first day.

At 1 p. m., the Association assembled at the hospital of Dr. Derwent and examined several very interesting and peculiar cases.

At 2 p. m, the meeting was called to order. The Secretary read several letters and telegrams expressing regret for necessary absence. Also a letter from W. S. Williams, President of the U.S.V.M.A., inviting this Association to take an active interest in the International meeting of the U.S.V.M.A., to be held in Chicago next September.

President M. E. Johnson, being absent, his address was read by the Secretary.

Prof. M. Stalker presented a report for the committee on legislation, detailing the obstacles the committee found in their endeavor to secure the enactment of an act regulating the practice of Veterinary Medicine and Surgery in Iowa. That it was finally necessary to have the bill killed in the senate committee in order to prevent the passage of a very undesirable law, about to be reported by said committee, which would legalize the uneducated practitioner and make the situation worse rather than better.

A vote of thanks was tendered the committee and the committee continued.

Dr. Edwards submitted a report for the committee on collective statistics, which was freely discussed by all present. Many interesting cases were reported verbally.

The Board of Censors reported favorably on the following named applicants for membership: H. M. Replogle, J. A. Replogle, W. R. Cooper, E. K. Paine, T. A. Shipley, S. H. Bauman, Davis, Coffee and N. Sorensen.

Upon motion the Secretary cast the ballot of the Association for the above named applicants.

The auditing committee reported the Treasurer's accounts correct.

The Secretary–Treasurer made a report in which he called attention to the itinerant habit of some of the graduates who locate in the State, and requested the members to notify him of all removals and new-comers. All members were urged to take an active interest in the Chicago meeting of the U.S.V.M.A., and the advantage of having a committee of arrangements to co-operate with the U.S.V.M.A. committee of arrangements was dwelt upon.

The financial report showed $47.00 in the treasury.

It was moved and carried that the President appoint a committee of arrangements for this Association at the Chicago meeting.

EVENING SESSION.

Dr. W. R. Cooper read a paper entitled "A Study of Septic Germs." Discussed by Drs. Stalker, Brown, T. A. Shipley, Stewart, Edwards.

Dr. L. U. Shipley read a paper entitled "Congestion of the Liver." Discussed by Drs. Stewart, Edwards, Brown, T. A. Shipley, Chatterton.

A sumptuous banquet in the cafe of the Tremont added much to the social pleasures of the meeting.

SECOND DAY.

10 a. m. Meeting called to order by Dr. Edwards.

Dr. G. L. Buffington read a paper entitled "Sclerostoma Tetracanthum." Discussed by Drs. Edwards, Brown, Stalker, Austin H. Shipley.

Dr. Reynolds read a paper, subject "Fistula." Discussed at great length by all the members present.

Adjourned for dinner.

1.30 p. m. Paper by Dr. W. B. Niles, entitled "Disinfectants in Veterinary Practice." Discussed by Drs. H. Shipley, Stewart, Reynolds, T. A. Shipley, Buffington and Derwent.

Paper by J. A. Replogle, entitled "Carbolic Acid as a Disinfectant," was the last paper read and discussed.

Paper, "Experiments with Lysol," was read by title only.

Election of officers resulted as follows: President, Dr. F. H. P. Edwards; 1st Vice-President, Dr. W. B. Niles; 2nd Vice-President, Dr. M. H. Reynolds. Secretary and Treasurer, S. Stewart.

Censors: Drs. J. E. Brown, E. E. Sayers, H. Shipley.

The President made the following appointments. Committee of Arrangements: Drs. J. E. Brown, S. Stewart and T. A. Shipley. Committee Collector Statistics: Drs. Wm. A. Starkey, M. Green and T. A. Bown.

On motion a vote of thanks was tendered the essayists for their valuable papers, also a vote of thanks to the Phoenix Club for the use of their rooms.

Adjourned.

S. STEWART, *Secretary.*

